# Computational Polarization Imaging In Vivo through Surgical Smoke Using Refined Polarization Difference

**DOI:** 10.1002/advs.202309998

**Published:** 2024-06-05

**Authors:** Daqian Wang, Jiawei Song, Jun Gao, Ji Qi, Daniel S. Elson

**Affiliations:** ^1^ Research Center for Frontier Fundamental Studies Zhejiang Lab Hangzhou 311121 China; ^2^ School of Computer and Information Hefei University of Technology Hefei 230601 China; ^3^ Hamlyn Centre for Robotic Surgery Imperial College London London SW7 2AZ UK; ^4^ Department of Surgery and Cancer Imperial College London London SW7 2AZ UK

**Keywords:** computational polarization imaging, in vivo, polarimetric endoscopic imaging system, refined polarization difference, surgical smoke removal

## Abstract

In surgery, the surgical smoke generated during tissue dissection and hemostasis can degrade the image quality, affecting tissue visibility and interfering with the further image processing. Developing reliable and interpretable computational imaging methods for restoring smoke‐affected surgical images is crucial, as typical image restoration methods relying on color‐texture information are insufficient. Here a computational polarization imaging method through surgical smoke is demonstrated, including a refined polarization difference estimation based on the discrete electric field direction, and a corresponding prior‐based estimation method, for better parameter estimation and image restoration performance. Results and analyses for ex vivo, the first in vivo animal experiments, and human oral cavity tests show that the proposed method achieves visibility restoration and color recovery of higher quality, and exhibits good generalization across diverse imaging scenarios with interpretability. The method is expected to enhance the precision, safety, and efficiency of advanced image‐guided and robotic surgery.

## Graphical abstract

A computational polarization image restoration method that exploits refined polarization difference is proposed and validated through in vivo animal experiments and human oral cavity tests, using a compatible polarization‐resolved endoscope system. The method demonstrates superiority in interpretability, generalizability, visibility, and color restoration, holding the potential to improve the precision, safety, and efficiency of surgical procedures.

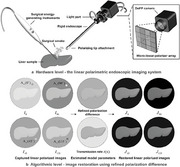

## 1 Introduction

Surgical smoke is produced by the interaction of tissue and energy‐generating instruments (for dissection and hemostasis).^[^ ^1^ ^]^ In some procedures (electrosurgery, laser surgery, high‐frequency electrocoagulation), the inevitable generation of surgical smoke can affect visibility and image quality,^[^ ^2^ ^]^ which leads to a range of problems including influencing the surgeon's operation and decision making, introducing potential risks to the surgery, limiting further image processing, etc. In endoscopic/laparoscopic surgery in particular, a large amount of smoke tends to accumulate in the enclosed cavity. Therefore, the integration of surgical smoke removal with existing endoscopic imaging systems is necessary.

Surgical smoke removal can be viewed as a visibility restoration problem, and existing smoke removal methods for surgical/endoscopic/laparoscopic images^[^ ^3^, ^4^, ^5^ ^]^ are mainly derived from visibility restoration methods. Generally, visibility restoration methods are divided into two categories: model‐based and data‐based methods. Model‐based methods are usually based on the image degradation model, with the parameters estimated according to priors, experimental phenomena or assumptions to achieve the recovery of the affected images. Representative methods include those based on the dark channel prior (DCP),^[^ ^6^ ^]^ haze line prior (HLP),^[^ ^7^ ^]^ color attenuation prior (CAP),^[^ ^8^ ^]^ polarization (POL),^[^ ^9^, ^10^, ^11^, ^12^, ^13^ ^]^ etc. These usually result in a visibility restoration effect and have the advantage of reduced processing time and computing resources, although the priors or assumptions may have limitations that affect the overall performance. Data‐based methods have developed rapidly including many recent network structures, e.g., the DehazeNet,^[^ ^14^ ^]^ MSCNN,^[^ ^15^ ^]^ FFA‐Net,^[^ ^16^ ^]^ DehazeFormer,^[^ ^17^ ^]^ C2PNet,^[^ ^18^ ^]^ DEA‐Net.^[^ ^19^ ^]^ They usually perform well but with poor interpretability, and the overall performance depends on the generalization ability of the network, the representativeness and completeness of the dataset and the sophistication of the computing resources.

However, for the solution of surgical smoke removal, the implementation of data‐based methods has certain difficulties, while model‐based approaches can be chosen as preferred solutions. Specifically: i) due to the ever‐changing surgical scenarios and conditions (different types of instruments, tissue, smoke distributions and illumination, etc.), establishing a comprehensive real‐world surgical smoke dataset (including smoke‐affected and corresponding smoke‐free images) is a complex task; ii) for a limited range of surgical scenarios, the tissue signal attenuation is no longer related to scene depth, but to the non‐uniform distribution of smoke (lack of a synthetic basis and random presentation), making it difficult to synthesize a realistic surgical smoke dataset; iii) most data‐based methods use synthetic datasets (including the ground truth of the model parameters used to generate the datasets) to train the network, therefore, these methods are often not compatible with real world datasets.

In addition to being able to restore visibility in scenarios such as the presence of haze,^[^ ^9^ ^]^ and imaging underwater^[^ ^10^, ^11^ ^]^ or through turbid liquids,^[^ ^12^, ^13^ ^]^ polarization imaging technology has been applied in the biomedical field,^[^ ^20^, ^21^, ^22^, ^23^, ^24^, ^25^ ^]^ and can be a potential approach for surgical smoke removal.^[^ ^26^ ^]^ Light polarization can be altered by scattering, reflection, refraction and birefringence during propagation in a medium. Through the analysis of the initial and output polarization states, the physical and optical properties of the medium can be represented and distinguished, which allows polarization imaging to achieve richer information than conventional optical imaging. The polarization‐based restoration method is basically based on an image degradation model, by solving a pair of orthogonal polarized channels minimally and maximally affected by the scattering medium, the parameters corresponding to target and scattered light can be estimated. However, existing polarization‐based restoration methods have shortcomings in parameter estimation, such as those that require artificially selected areas, multiple measurements, recalibration of parameters, or associated with various image processing operators, such as mutual information^[^ ^11^ ^]^ and scene restoration algorithms.^[^ ^12^ ^]^ Such processing may affect the accuracy of the restoration, in addition to interpretability issues.

**Figure 1:**
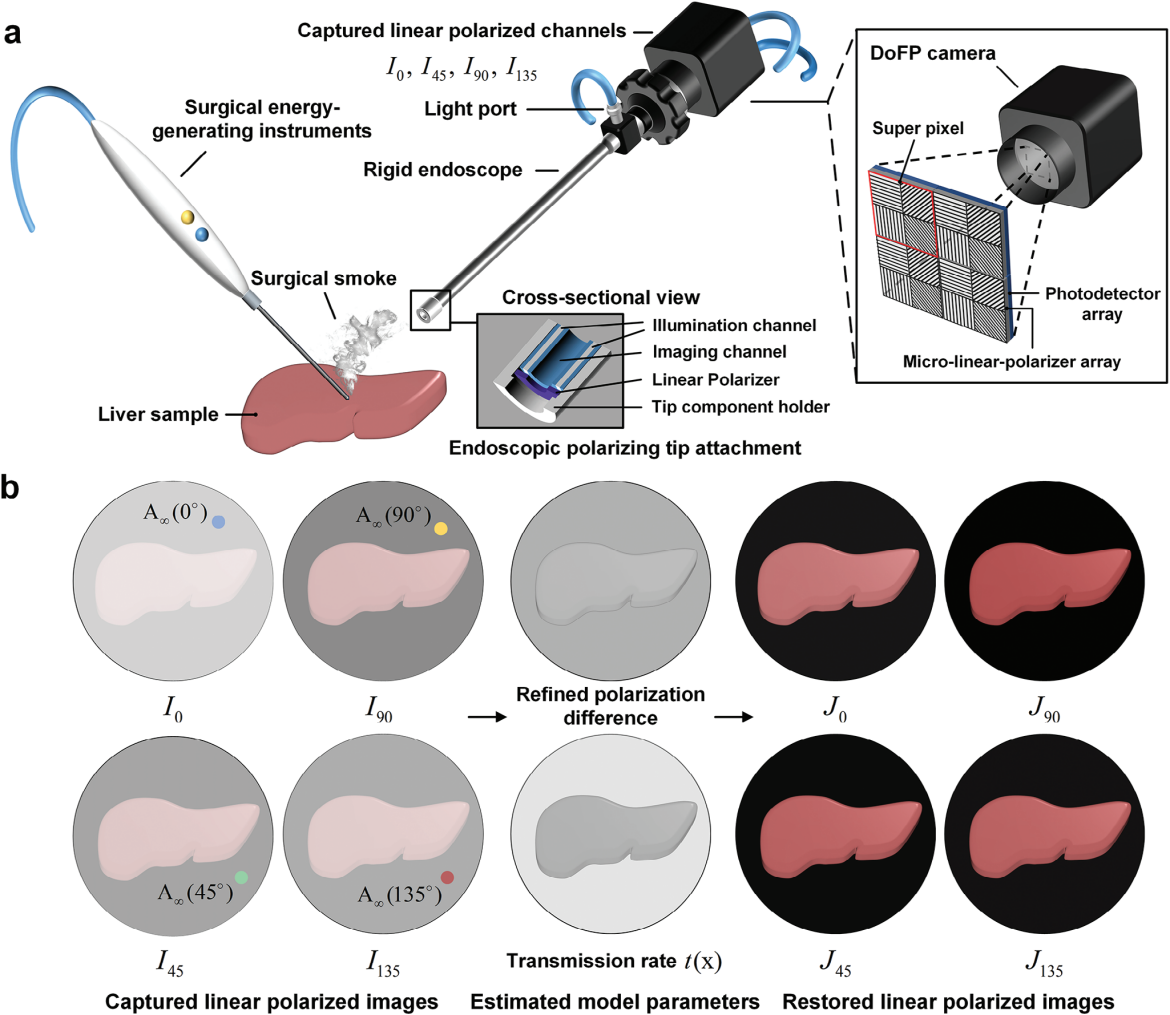
Computational polarization imaging for surgical smoke removal during surgical endoscopy. a) The linear polarimetric endoscopic system. The illumination is linearly polarized by attaching a linear polarizer at the tip of the illumination channel of the system. A polarization maintaining surgical endoscope and a division‐of‐focal‐plane (DoFP) linear polarimetric camera were used to capture four linearly polarized images (*I*_0_,*I*_45_,*I*_90_,*I*_135_) of biological tissues. The imaging scenario is simplified as a bi‐layer scattering medium, comprising an upper layer of smoke and a deeper layer of tissue. b) The proposed refined PD estimation method, which could be used to improve parameter estimation accuracy of polarization‐based image degradation model, based on four captured linear polarized images. The estimated parameters *t*(x) (transmission rate) and A_∞_ (the intensity of ambient light with infinite optical depth) are incorporated into the model, resulting in improved channel‐by‐channel image restoration.

In this paper, we propose a restoration method based on refined polarization difference (PD) calculation for surgical smoke images, and validate the method in the ex vivo, and in vivo experiments with a linear polarimetric endoscopic imaging system for different imaging conditions, as shown in **Figure** **1**. Based on the polarization‐based image degradation model, our method includes a refined PD for parameter estimation and a prior‐based real‐time PD estimation method. Qualitative and quantitative comparisons demonstrated that, i) our method outperforms those existing model‐based and data‐based methods in terms of interpretability, visibility restoration and color recovery; ii) our method has good generalizability performance, which has been validated in the scenarios with different types of scattering media (generated by a “fogger” system, cigarettes or “simulated” surgical smoke through cutting the fat tissue) and different targets (Spydercheckr test card, the USAF‐1951 Resolution Target, ex vivo and in vivo tissues); iii) our method holds the potential to be clinically translated and is able to help surgeons operate more precisely, quickly, and securely in minimally invasive surgery.

## 2 Results

### 2.1 Polarization Difference for Model Parameter Estimation

For active linear polarimetric imaging, the intensity differences between captured polarized channels can provide additional information and a physical basis for subsequent processing. When imaging through scattering media, the captured *I_co_* (*I*_0_, co‐polarized intensity) and *I*_*c* *r* *o*_ (*I*_90_, cross‐polarized intensity) can be regarded as approximately the polarization channels most and least affected by the backscattered component (i.e., smoke component), as been proved through simulations and experiments.^[^ ^12^, ^13^, ^26^ ^]^ Therefore, on the basis of the polarization‐based image degradation model, once the A_∞_ (the intensity of ambient light with infinite optical depth) has been estimated,^[^ ^27^ ^]^ the transmission rate *t*(x) can be estimated as:

(1) $$t\left(x\right)=1-\frac{I_{\mathrm{co}}\left(x\right)-I_{\mathrm{cro}}\left(x\right)}{A_{\infty }\left(\mathrm{co}\right)}=1-\frac{\mathrm{PD}(x)}{A_{\infty }\left(\mathrm{co}\right)}$$

where the term “x” implies the need for pixel‐wise estimation. The detailed derivation can be found in Section 5.1. Equation 1 has been proved to efficiently estimate the backscattered component (by PD) in media with low scattering coefficients.^[^ ^26^ ^]^ However, the proximity between PD and real backscattered component will determine the restoration performance. When the smoke density increases, the PD estimation is compromised for strongly scattering media (in terms of accuracy in estimating backscattered component), because the image field is dominated by polarized light with random polarization state (due to multiple scattering), and the orthogonal polarization is partially affected by the backscattered component. Therefore, the inaccurate estimation of the backscattered component by PD affects the model parameter estimation accuracy and smoke removal performance. The study of reasonable processing method to achieve more accurate parameter estimation is the focus of this paper.

### 2.2 Refined PD

The purpose of varying the PD estimation method is to improve the accuracy of the parameter estimation and processing. One possible idea is to analyze the composition of the captured “superimposed” polarization intensities from the perspective of the polarization acquisition mechanism. Since Malus’ law can simply and directly characterize the intensity variation of a beam of polarized light (with specific electric field direction) after passing through a polarizer, therefore, the composition of captured polarization intensities can be analyzed from the perspective of Malus’ law. In an imaging environment with the polarized light in different electric field directions, the intensity transmitted by a polarizer (axis at direction *θ*_0_) can be approximated as the sum of “projected” components from different electric field directions:

(2) $$I_{\theta_{0}}=\sum_{\theta =0^{\circ }}^{360^{\circ }}S\left(\theta \right)\cdot p_{\theta_{0}}\left(\theta \right)$$

where *S*(*θ*) is the intensity of a specific electric field interval *θ*, $p_{\theta_{0}}\left(\theta \right)=\cos^{2}(\theta_{0}-\theta )$ is the “weight vector” and describes the contribution of each *S*(*θ*) element into the overall transmitted intensity $I_{\theta_{0}}$ by a polarizer.

Theoretically, if the initial polarization direction is aligned at 0°, the intensity of polarized light with 90° electric field direction *S*(90°) contains the lowest backscattered component. Therefore, if it is possible to obtain the values of *S*(*θ*), the PD could be “accurately” obtained by the polarization difference between the intensity of each electric field interval, the intensity of 90° electric field interval and the corresponding weight:

(3) $$\begin{array}{ccc} PD^{\prime } & = & \sum_{\theta =0^{\circ }}^{360^{\circ }}\left(S\left(\theta \right)-S\left(90^{\circ }\right)\right)\cdot \cos^{2}\theta \\ & = & I_{0}-180\cdot S\left(90^{\circ }\right) \end{array}$$

However, estimating the intensity of the electric field at different polarization intervals (from any captured polarization measurements) is complex, thus an alternative form in Equation 3 indicates that the refined PD can be estimated only by the intensity of *I*_0_ and *S*(90°), which significantly reduces the computational and time cost of the estimation. The second term is related to the polarization density at the orthogonal state, i.e., an “ideal orthogonal polarized channel”, and is generated by replacing each of the discrete electric vector intensities in *I*_90_ with *S*(90°). Then the refined PD was used to replace the direct PD in Equation 1 to improve estimation accuracy of the backscattered component and the transmission rate *t*(x).

### 2.3 Refined PD Estimation Based on Prior Information

To meet application requirements, the refined PD should be estimated in real‐time. However, direct calculation of the intensities of discrete electric field directions is impractical and time‐consuming, as well as being difficult to estimate in real‐world experiments. It is more practical and feasible to obtain prior information, such as the distribution of discrete electric intensities, through Polarized Monte Carlo simulations where the exact polarization state can be tracked, in order to develop a prior‐based refined PD estimation method. The introduction to Polarized Monte Carlo modeling^[^ ^28^, ^29^, ^30^, ^31^, ^32^, ^33^, ^34^, ^35^, ^36^, ^37^ ^]^ can be found in the Supporting Information.

**Figure 2:**
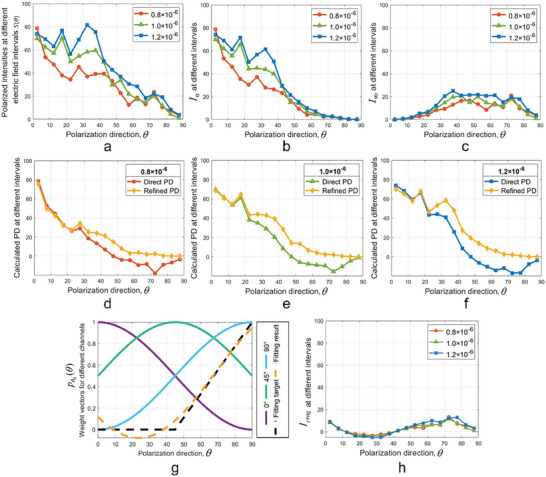
Variation of simulated distributions with *θ*. a) *S*(*θ*), b) *I*_0_, c) *I*_90_; direct PD and refined PD for densities (*n*/µm^3^): d) 0.8 × 10^‐6^, e) 1.0 × 10^‐6^, and f) 1.2 × 10^‐6^; g) The distribution of $p_{\theta_{0}}\left(\theta \right)$ when *θ*_0_ was at 0°, 45° and 90°, respectively, and one suitable target curve *p_T_* (*θ*) (black dashed line) and the fitting is achieved with one possible result *p_F_* (*θ*) (orange dashed line); the simulated distribution of “clearer” orthogonal polarization h) *I_crog_*, *I_crog_* could reduce more backscattered component compared with c) *I*_90_.

A group of simulation results for a large particle medium with three different densities (corresponding scattering coefficients: 0.50, 0.62, 0.75 cm^‐1^) are shown in **Figure** **2**, and the implementation details and parameter settings^[^ ^38^, ^39^, ^40^, ^41^, ^42^, ^43^ ^]^ are shown in the Experimental Section. The captured intensity of each polarization direction was regarded as a superposition of the intensity of each discrete electric field interval (5°, in order to balance sampling accuracy and computational efficiency), and these discrete points were connected by lines to show the variation trend. Each discrete point of the *S*(*θ*) distribution represented the intensity of photons (the central intensity of the captured *I* channel) falling into the corresponding interval, as shown in Figure 2a. *S*(*θ*) showed an overall downward trend with the increase of *θ*, and as the scattering coefficient increased, the distribution of *S*(*θ*) became more discretized. *I*_0_ and *I*_90_ were calculated in each interval, as shown in Figure 2b,c. The direct PD and refined PD in each interval for different densities are shown in Figure 2d–f. Compared with the direct PD, the refined PD could be used to better estimate the backscattered component, particularly for the medium with larger scattering coefficient. For active linear polarimetric imaging, in the range of 0° to 90°, the distribution of $p_{\theta_{0}}\left(\theta \right)$ for certain directions (0°, 45° and 90°) is shown by the solid line in Figure 2g.

The estimation of *S*(90°) was the focus of the refined PD estimation (Equation 3) and a prior‐based estimation method was proposed. According to the simulations, the *S*(90°) component was completely maintained in *I*_90_, as shown in Figure 2a,c, however, if *S*(90°) was estimated directly from *I*_90_, the result would probably be affected due to the existence of backscattered component, especially for strongly scattering media. Therefore, a preprocessing step was added in order to reduce the impact of the backscattered component before estimation. One possible solution was to artificially construct a weight vector (target) curve (*p_T_* (*θ*), black dashed line) in place of *p*_90_(*θ*) (blue solid line), as shown in Figure 2g, thus generating a “clearer” orthogonal polarization according to Equation 2 to eliminate the intensity in the electric field interval where the backscattered component was mainly distributed. So it was transformed into a curve fitting problem, and the base curves for the fitting were a series of $p_{\theta_{0}}\left(\theta \right)$ with variable amplitude, variable phase but fixed frequency, including *p*_0_(*θ*), *p*_45_(*θ*), *p*_90_(*θ*) in Figure 2g. The fitting result *p_F_* (*θ*) is shown by the orange dashed line in Figure 2g and we could use *p_F_* (*θ*) to replace *p*_90_(*θ*) for generating a “clearer” orthogonal polarization *I_crog_*. Then, *S*(90°) was estimated based on the uniform distribution of the generated *I_crog_*, as shown in Figure 2h. The implementation details of *S*(90°) and refined PD estimation are shown in Experimental Section. Once the refined PD was estimated and used to replace the direct PD in Equation 1, the image restoration method based on the refined PD could be achieved.

### 2.4 Ex Vivo Experiment

**Figure 3:**
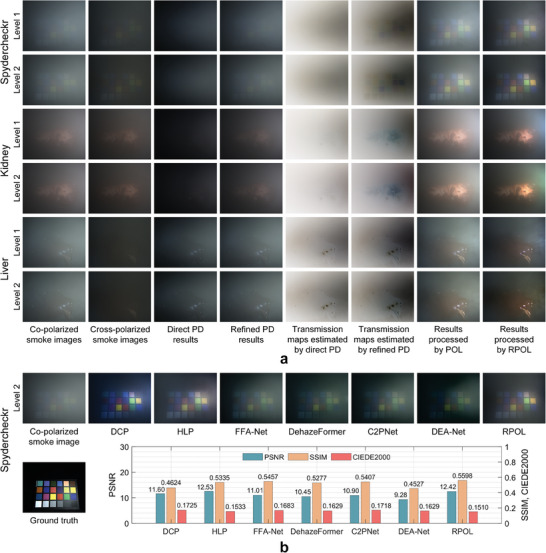
Smoke removal results for test target and ex vivo tissue samples. a) Co‐polarized, cross‐polarized smoke images, the parameters and results processed by the direct PD and refined PD under two different smoke density levels (Level 1 was denser) from a SpyderCheckr, a porcine kidney and a liver sample; b) Comparison of smoke removal results for different methods: DCP, HLP, FFA‐Net, DehazeFormer, C2PNet, DEA‐Net, and our method RPOL, with further quantitative results: SSIM, PSNR (the higher the better) and CIEDE2000 (the lower the better).

We conducted experiments for a SpyderCheckr test target and ex vivo tissue samples (porcine kidney and liver) on desktop linear polarimetric imaging system, in order to preliminarily validate our method from the perspective of smoke removal and color restoration, as shown in **Figure** **3**. The scenario was filled with high density smoke produced by a fogger and the sample was almost completely obscured by smoke. The incident polarization state was set to horizontal linear polarized (0°) and the polarized smoke images were collected under different smoke density levels. The intermediate variables generated during calculation, including the estimated direct PD and refined PD, the estimated transmission maps and restored results processed by POL and RPOL are shown in Figure 3a.

Through qualitative comparison, the overall intensity of the refined PD result was higher than that of the direct PD result, which proved that the refined PD can estimate the backscattered component more completely. Similarly, the transmission maps estimated by the refined PD had overall lower intensity (more “darker” pixels) than those estimated by the direct PD (the lower the transmission rate, the better the restoration performance to be expected). In addition, the advantages of the refined PD were also intuitively reflected in the processed smoke removal results, where the results processed by RPOL had better image visibility and richer details than the results processed by POL, as shown in Figure 3a.

A group of comparative experiments for the SpyderCheckr sample between different restoration methods is shown in Figure 3b. We processed smoke images in different polarization directions and superimposed the processed co‐polarized and cross polarized images (according to the definition of Stokes parameters) for better qualitative analysis. The full‐reference image quality assessment indicators PSNR (peak signal‐to‐noise ratio), SSIM (structure similarity index measure) and CIEDE2000 (CIE 2000 color difference formula) were adopted for the quantitative analysis. It could be observed that all methods reduced the impact of smoke. Although the DCP method was effective in smoke removal, it underestimated the transmission parameters, leading to a low luminance, oversaturation effect, and information loss. The HLP method could remove the smoke to a considerable extent, however, the color restoration was not accurate enough in some scenarios through qualitative and quantitative analysis, and a small amount of smoke still existed in the processed results. Four pre‐trained models of data‐based methods, FFA‐Net (OTS), DehazeFormer (Ourdoor ‐m), C2PNet (OTS) and DEA‐Net (OTS), had generalization effect and could restore both visibility and color, however, the smoke removal was still not thorough enough. Our proposed method RPOL could reduce the impact of smoke and restore the original color well.

### 2.5 In Vivo Mouse Experiment

We conducted the first in vivo smoke removal experiments, using mouse tissue as the target, and paired with a polarization maintaining endoscopic imaging system,^[^ ^22^ ^]^ a laparoscopic simulator and surgical instruments, which enabled us to test the effectiveness of our method in a way that was closer to clinical trials. Ethical approval was obtained from Zhejiang Lab Ethics Committee (reference number ZJSL‐2022‐9). It was worth emphasizing that we avoided color correction operations, but instead processed the captured intensities and evaluated the processed results to ensure the correctness of the polarization information distribution.

**Figure 4:**
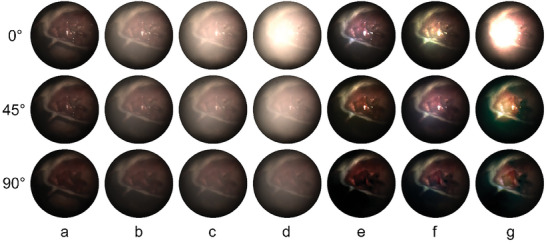
Comparison of restoration results under different smoke density levels. 0°, 45°, 90° a) Polarized ground truth, polarized smoke images under b) Level 1, c) Level 2, and d) Level 3; restored results under e) Level 1, f) Level 2, and g) Level 3. Level 1 was mild density, Level 2 was moderate and Level 3 was extremely heavy smoke.

First, in order to provide a reference for quantitative evaluation, we generated smoke by cutting a piece of pork adipose tissue while preserving the integrity of the mouse sample, and covered the entire field of view with smoke. The processed result indicated that our method was able to eliminate the smoke from the images and enhance the structure contrast for different smoke density levels, as shown in **Figure** **4**. Specifically, the tissue contours of the mouse in the smoke image were progressively less clear as the smoke density increased, as shown in Figure 4b–d, while the corresponding restored results showed that organs and contours in the mouse abdominal cavity could be more clearly distinguished, as shown in Figure 4e,f. It should be emphasised that polarimetric endoscopy had an incomparable advantage over conventional white‐light endoscopy in dealing with scattered light interference during surgery. When the smoke density was too high, the 0° polarized image had a large overexposed area due to the strong backscattering, resulting in a serious loss of image information, as shown in Figure 4d, whereas the 45° and 90° polarized images could eliminate the backscattered light to varying degrees. Therefore, the restored 45° and 90° images were still able to provide reference information, as shown in Figure 4g.

**Figure 5:**
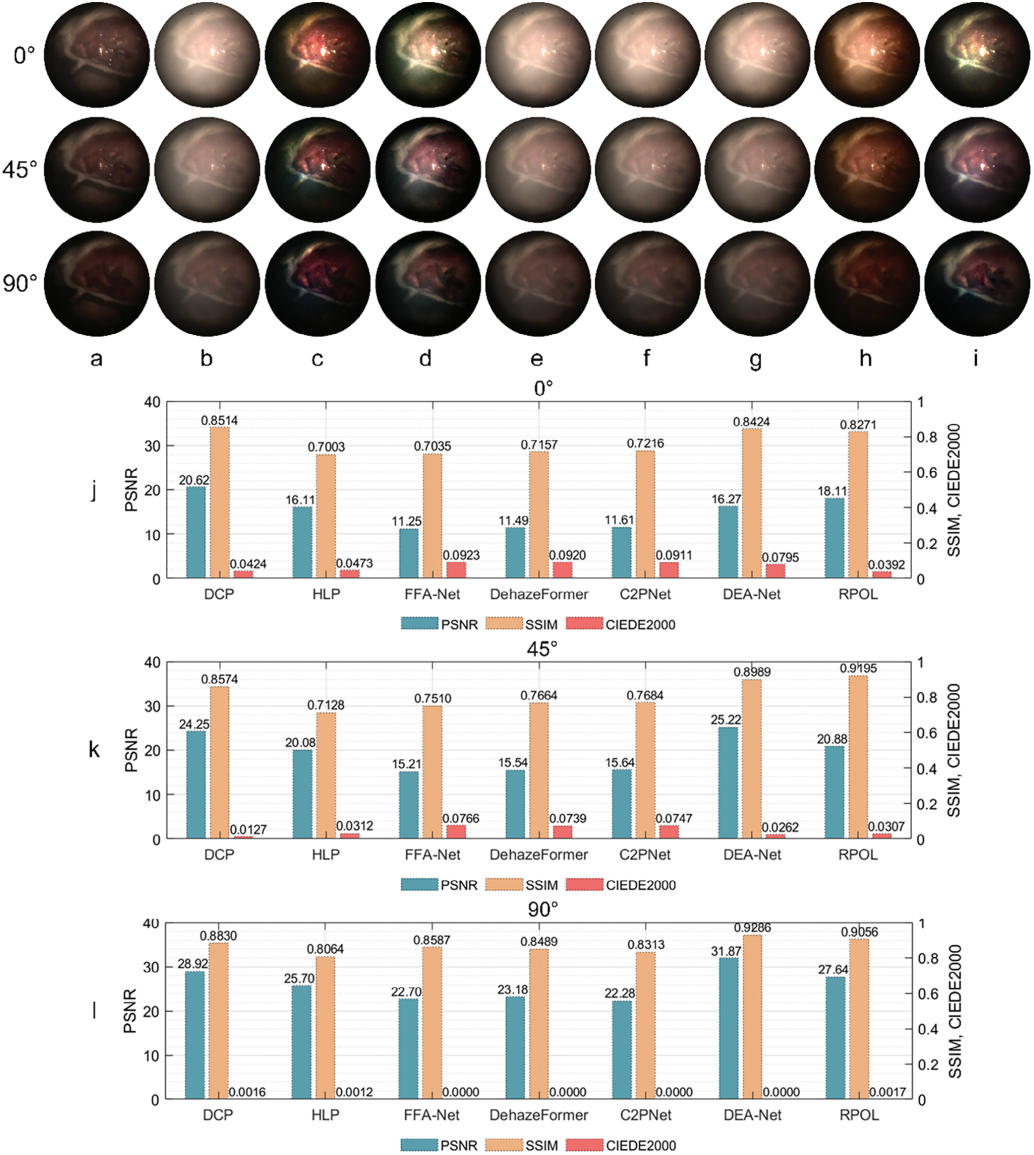
Comparison of smoke removal results for different methods (density level 2). a) Ground truth, b) 0°, 45°, 90° polarized smoke images, and co‐polarized results processed by c) DCP, d) HLP, e) FFA‐Net, f) DehazeFormer, g) C2PNet, h) DEA‐Net, and i) RPOL, with further quantitative results in j–l) SSIM, PSNR (the higher the better) and CIEDE2000 (the lower the better).

A group of comparative experiments for different methods indicated that all methods worked better for polarized images in the 90° direction (since orthogonal polarized channel was partially influenced by the scattering medium), however, for the more affected 0° and 45° polarized channels, the traditional model‐based methods had significant smoke removal performance, while some of the data‐based pre‐trained models had limited generalization capabilities, as shown in **Figure** **5**a–i and further quantified in Figure 5j–l. Specifically, the DCP method had very thorough smoke removal but was accompanied by oversaturation. The HLP method also achieved good results, but a small amount of smoke was still visible and the color restoration was not accurate enough. For the data‐based methods, in contrast to the previous set of experiment (Figure 3), the pre‐trained models of FFA's OTS, DehazeFormer's outdoor ‐m and C2PNet's OTS were not suitable for this experimental scenario, as the performance of smoke removal was not very obvious, as shown in Figure 5e–g. While DEA‐Net's OTS was also less effective, but its HAZE4K performed well, as shown in Figure 5h, although for high densities it was slightly less effective. The proposed method RPOL had a thorough smoke removal and structural enhancing performance for different smoke densities, and did not suffer from oversaturation, but there was a slight color distortion in the processed 0° polarized image, as shown in Figure 5i.

**Figure 6:**
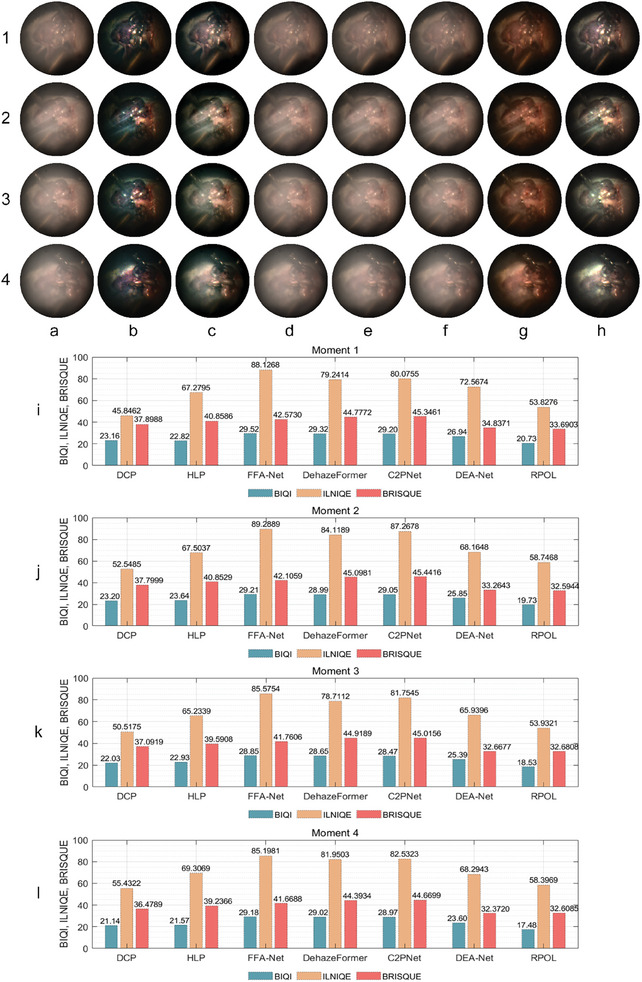
Comparison of smoke removal results for four different image fields with surgical tools (the electric soldering iron, the needle holder and scissors) for different methods. a) 0° polarized smoke images, and results processed by b) DCP, c) HLP, d) FFA‐Net, e) DehazeFormer, f) C2PNet, g) DEA‐Net, and h) RPOL, with further quantitative results in i–l) BIQI, ILNIQE, and BRISQUE (the lower the better).

The second round of experiments involved processing polarized smoke images of the mouse sample with surgical tools, as shown in **Figure** **6**, in order to validate whether surgical instruments could be effectively identified from tissues after processing, which is very important for precise operation during surgery. Because the manipulation of the surgical tools caused the scenario to change constantly during the “simulated surgery,” which prevented us from obtaining the corresponding ground truth for quantitative evaluation. Therefore, the non‐reference image quality assessment indicators blind image quality index (BIQI), integrated local natural image quality evaluator (ILNIQE), and blind/referenceless image spatial quality evaluator (BRISQUE) were adopted for the quantitative analysis. Generally, the conclusions were similar to those of the previous group. DCP had a thorough smoke removal, but oversaturation and color distortion were severe, and the distinction between surgical instruments and mouse tissue was not clear enough, perhaps leading to misjudgment in surgical decisions. HLP had good smoke removal results, but did not perform well at removing dense smoke. FFA's OTS, DehazeFormer's outdoor ‐m and C2PNet's OTS had limited generalization ability for this scene, where a large amount of smoke component still existed in the processing results. The DEA‐Net's HAZE4K had some recovery effect, but the color reproduction was not accurate enough. In comparison, the proposed method RPOL had a thorough smoke removal performance (better than HLP), had a more accurate color restoration, and could better show the distinction between surgical tools and mouse tissues, thus having the potential to be applied to real surgical smoke scenarios. However, it was worth emphasizing that these non‐reference quantitative metrics may not fully reflect the effectiveness of image restoration, the accuracy of color restoration and the agreement with the ground truth, which may therefore deviate from the qualitative analysis.

### 2.6 In Vivo Oral Experiment

**Figure 7:**
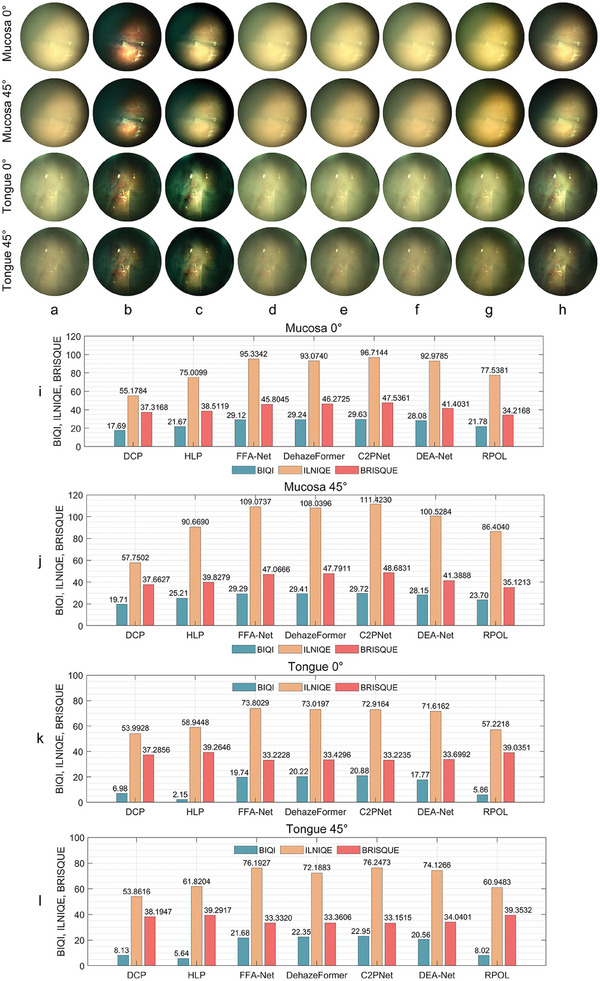
Comparison of smoke removal results (oral cavity tissue) for different methods. a) 0° or 45° polarized smoke images, and results processed by b) DCP, c) HLP, d) FFA‐Net, e) DehazeFormer, f) C2PNet, g) DEA‐Net, and h) RPOL, with further quantitative results in i–l) BIQI, ILNIQE, and BRISQUE (the lower the better).

Due to disparities between human and animal tissues in terms of tissue texture, movement patterns, and the absorption and scattering properties of light, we conducted imaging experiments within the human oral cavity, with the smoke produced by cigarette smoking, in order to further validate the generalizability and applicability of the method. Ethical approval for this experiment was obtained from Zhejiang Lab Ethics Committee (reference number ZJSL‐2022‐9). Similarly, we avoided the color correction operation and instead processed and evaluated the captured information directly. A group of comparative experiments between different smoke removal methods is shown in **Figure** **7**. The results indicated that the model‐based method performed better than the data‐based pre‐trained model. The DCP method “over‐processed” the image: although the smoke removal effect was obvious, the overall color distortion was serious. The HLP method had better results, but the color reproduction of the tongue tissue was not accurate enough, which resulted in an overall greenish display. The data‐based FFA's OTS, DehazeFormer's outdoor ‐m, C2PNet's OTS and DEA‐Net's HAZE4K did not have obvious smoke removal performance, as shown in Figure 7d–g. Our method was close to the performance of the HLP method for smoke removal, but was more accurate for color recovery of tongue tissue, as shown in Figure 7h.

Given surgeons’ imperative to discern and identify intricate structures like blood vessels, nerves, and tissue boundaries during surgery for accurate positioning and manipulation, the validation and evaluation of the restored spatial resolution of surgical smoke images become paramount. We further carried out a validation experiment to showcase the enhancement in spatial resolution achieved by the proposed method, using a USAF‐1951 Resolution Target, as illustrated in Section S4 of the Supporting Information.

## 3 Discussion

Surgical smoke generated during surgery can significantly impair visibility, impacting surgical procedures and safety, so addressing the issue of visibility restoration in surgical imaging is crucial. The integration of computational imaging techniques and the optimization of both hardware and algorithms can substantially enhance imaging quality. In particular, the acquisition of intensities across various polarization states can be considered as encoding of information at the hardware level, which is fundamental to achieving superior outcomes. Our research contributes at the algorithmic level by innovating the decoding process. This innovation primarily seeks to improve the precision of processing and utilizing the captured polarization data, while simultaneously ensuring both universality and interpretability.

Incorporating prior knowledge from Polarized Monte Carlo simulations has been found to be beneficial. Generally, polarization imaging is influenced by the properties of smoke, the imaging target and the illumination, certain prior information may be challenging to ascertain and validate through real experiments due to uncontrollable variables such as particle size and density. Therefore, the prior information obtained from simulations (i.e., the intensity distribution of *S*(*θ*), captured polarized intensities at different intervals) could provide a theoretical basis for the refinement of the PD estimation.

Upon applying the proposed method for smoky image restoration, presenting the enhanced images to clinicians via advanced technologies such as augmented reality (AR) and heads‐up displays (HUDs) should be beneficial.^[^ ^44^ ^]^ This can facilitate real‐time visualization of anatomical structures and surgical instruments that are otherwise obscured by surgical smoke, thereby enabling clinicians to pinpoint targets and execute precise manoeuvres with greater accuracy. Moreover, our method may mitigate the challenges associated with suboptimal 3D reconstruction due to surgical smoke, consequently improving the efficiency of AR.

The proposed method still has shortcomings, for example, when estimating the parameters of polarization‐based image degradation model, the polarization characteristics of the target was not considered (i.e., *J_co_* (x) ≈ *J_cro_* (x)). The captured polarization intensity consists of components from the target and ambient light, making it difficult to fully estimate the parameters of one component while eliminating the influence of the other. In general, for imaging targets such as tissues, disregarding the polarization characteristics could have minimal impact,^[^ ^26^ ^]^ while for strong specular reflection targets (e.g., surgical instruments), the restoration can perhaps be affected. Nevertheless, although obtaining the accurate polarization characteristics of the target (i.e., *J_co_* (x) − *J_cro_* (x)) is difficult, one may approximate it by minimizing the impact of the scattered ambient light *A* through the generation of synthetic co‐polarized and cross‐polarized intensities (i.e., *I_crog_*), guided by prior information (i.e., the distribution of *S*(*θ*)).

Besides surgery, the proposed image restoration method holds promise for application to a variety of scenarios involving scattering media, such as dusty atmospheres and underwater conditions. Although differences in imaging conditions result in variations in the distribution of captured polarization intensities, there are similarities in polarization transmission characteristics, such as the effectiveness of PD, or the distribution of *S*(*θ*). The validity of the prior information obtained from simulations across various scenarios is crucial for ensuring the generalizability of our method. When extending the proposed method to other application scenarios, the following steps can be followed: i) Identifying the medium conditions in the application scenario, including particle size distribution, anisotropy, relative refractive index, etc.; ii) Conducting Polarized Monte Carlo simulation experiments based on the medium conditions to obtain the polarization transmission characteristics and mastering prior knowledge. This process also aids in determining optimal polarization illumination conditions (i.e., the wavelength, intensity, polarization state) and imaging conditions (i.e., the setting of imaging system and imaging distance); iii) Conducting method testing based on the prior knowledge and adjusting the parameter estimation methods (such as the estimation of *S*(90°)), if necessary, to improve the imaging performance.

## 4 Conclusion

An endoscopic image restoration technique based on computational polarization imaging has been demonstrated to improve the image quality affected by surgical smoke. Based on the polarization‐based image degradation model, we investigated the idea of decomposing the captured polarized intensities in discrete electric field directions, and proposed a refined PD to promote the “precision” of polarization information processing (compared to the general direct PD). Additionally, we developed a prior‐based method for real‐time refined PD estimation, subsequently developing an image restoration method based on refined PD. Our methodology was implemented on a linear polarimetric endoscopic imaging system and validation experiments including the first in vivo animal experiments and human oral cavity tests were conducted. Our results demonstrate that the proposed method outperforms those existing model‐based and data‐based methods in terms of interpretability, generalizability, visibility restoration and color recovery, and holds the potential to be clinically translated. We believe that this method could help surgeons to operate more precisely, quickly, and securely.

## 5 Experimental Section

### Polarization‐Based Image Degradation Model

Polarization‐based image degradation model is used to characterize the intensity composition of captured polarized light after propagation in mixed media. Generally, the captured intensities can be divided into the captured attenuated intensity from the target *D* and scattered ambient light *A*, respectively:

(4) $$I\left(x\right)=D\left(x\right)+A\left(x\right)=J\left(x\right)t\left(x\right)+A_{\infty }\left(1-t\left(x\right)\right)$$

where *I* is the captured intensity affected by the scattering media, *t* and A_∞_ are the parameters to be estimated, *J* describes the target intensity to be recovered, and x is a pixel in the image, which indicated that the corresponding parameter should be estimated pixel by pixel. In medical imaging, the depth of the local area is approximately the same, the definitions of *t* and A_∞_ are independent of the depth, but determined by the optical depth of the medium (e.g., smoke). The transmission rate *t* describes the proportion of radiance attenuation, which is determined by the optical depth *τ* of the local medium (i.e., the higher the density of medium, the lower the transmission).^[^ ^26^ ^]^ A_∞_ is a constant and is defined as the intensity of ambient light with infinite optical depth (i.e., the captured intensity when the target signal is completely obscured by scattering media).

For active linear polarimetric imaging, the captured *I_co_* (x) (co‐polarized intensity) and *I*_*c* *r* *o*_(x) (cross‐polarized intensity) can be regarded as approximately the most and least affected polarized channel by the backscattered component,^[^ ^12^, ^13^, ^26^ ^]^ therefore the polarization difference (PD) can be implemented to estimate the backscattered component:

(5) $$PD(x)=I_{co}\left(x\right)-I_{cro}\left(x\right)\approx A\left(x\right)$$

In addition, since the surface layer (smoke layer) contains a large number of polarization‐maintaining components, the backscattered component exists mainly in the co‐polarized channel (same direction as the incident polarization):

(6) $$A\left(x\right)=A_{\infty }\left(1-t\left(x\right)\right)=A_{\infty }\left(\mathrm{co}\right)\left(1-t\left(x\right)\right)$$

Therefore, the transmission rate *t*(x) can be estimated as:

(7) $$t\left(x\right)=1-\frac{I_{\mathrm{co}}\left(x\right)-I_{\mathrm{cro}}\left(x\right)}{A_{\infty }\left(\mathrm{co}\right)}=1-\frac{\mathrm{PD}(x)}{A_{\infty }\left(\mathrm{co}\right)}$$

Once A_∞_ was estimated,^[^ ^27^ ^]^ the smoke removal result *J*, and each specific restored polarized intensity *J_pol_* can be expressed as:

(8) $$\left\{\begin{array}{c} J\left(x\right)=\frac{I_{co}(x)+I_{cro}\left(x\right)-A_{\infty }\left(co\right)}{t\left(x\right)}+A_{\infty }\left(co\right)\\ J_{pol}\left(x\right)=\frac{I_{pol}\left(x\right)-A_{\infty }\left(pol\right)}{t\left(x\right)}+A_{\infty }\left(pol\right) \end{array}\right.$$

Existing research indicates that circular polarization imaging has advantages over linear polarization imaging in scattering media with large‐sized particles, including longer propagation distances, better imaging contrast and better polarization maintaining property.^[^ ^45^, ^46^ ^]^ The decision to avoid circular polarization arises from the phenomenon of helicity flip during its propagation in a medium, leading to a highly susceptible polarization state distribution influenced by environmental factors. This introduces uncertainties when predicting circular polarization transmission characteristics, and the performance of polarization difference.^[^ ^45^ ^]^ Additionally, compared to linear polarization imaging, circular polarization requires the addition of extra quarter‐wave plates for polarization generation and analysis, thus complicating the equipment and may potentially introducing additional errors.

### Composition of Captured Polarized Intensities

Understanding the composition of the captured intensity after passing through a polarizer is fundamental to accurate polarization information processing, and the same expression can be derived from the perspective of i) Malus’ law and ii) Stokes vector calculation, respectively.

i) Since Malus’ law describes the intensity of an ideal beam of linear polarized light after passing through a polarizer, when polarized light with different electric field directions is generated in the environment due to multiple scattering, the intensity transmitted by a polarizer (axis at direction *θ*_0_) can be approximated as the sum of “projected” components from different electric field directions:

(9) $$\begin{array}{ccc} I_{\theta_{0}} & = & \sum_{\theta =0^{\circ }}^{360^{\circ }}S\left(\theta \right)\cdot \cos^{2}(\theta_{0}-\theta )\\ & = & \sum_{n\Delta \sigma =0^{\circ }}^{360^{\circ }}S\left(n\Delta \sigma \right)\cdot \cos^{2}(\theta_{0}-n\Delta \sigma ) \end{array}$$

where Δ*σ* is the sampling interval division for electric field directions, *n* denotes the *n*th sampling interval. The concept of sampling was introduced because when actually decomposing the captured polarized intensity in the direction of discrete electric field vectors, it is necessary to choose a suitable sampling interval and number so as to achieve a balance between accuracy and efficiency. The smaller the Δ*σ*, the more accurate the calculation result, but the higher the computation cost. The intensity of a specific sampling interval can be expressed as:

(10) $$S\left(\Delta \sigma \right)=\int_{\Delta \sigma }p(\Delta \sigma )d\Delta \sigma$$

where *p*(Δ*σ*) is the probability density function of photon energy distribution in an interval.

ii) Setting the reference direction to 0°, if the polarizer is fixed at *θ*_0_, the Mueller matrix of an ideal polarizer is:

(11) $$M_{p}\left(\theta_{0}\right)=\frac{1}{2}\left[\begin{array}{cccc} 1 & \cos2\theta_{0} & \sin2\theta_{0} & 0\\ \cos2\theta_{0} & \cos^{2}2\theta_{0} & \cos2\theta_{0}\sin2\theta_{0} & 0\\ \sin2\theta_{0} & \cos2\theta_{0}\sin2\theta_{0} & \sin^{2}2\theta_{0} & 0\\ 0 & 0 & 0 & 0 \end{array}\right]$$

For polarized light with different electric field directions, the total Stokes vector was the sum of the product of every photon (in each sampling interval) normalized Stokes vector and weight:

(12) $$\begin{array}{ccc} SP_{\mathrm{interval}} & = & \sum_{n\Delta \sigma =0^{\circ }}^{360^{\circ }}SP(n\Delta \sigma )=\sum_{n\Delta \sigma =0^{\circ }}^{360^{\circ }}S\left(n\Delta \sigma \right)\left[\begin{array}{c} 1\\ \cos2n\Delta \sigma \\ \sin2n\Delta \sigma \\ 0 \end{array}\right]\\ & = & \sum_{\theta =0^{\circ }}^{360^{\circ }}S\left(\theta \right)\left[\begin{array}{c} 1\\ \cos2\theta \\ \sin2\theta \\ 0 \end{array}\right] \end{array}$$

When the polarized light with different electric field directions transmitted by a polarizer, the captured Stokes vector is:

(13) $$\begin{array}{c} \begin{array}{ccc} S_{\theta_{0}} & = & M_{p}\left(\theta_{0}\right)SP_{\mathrm{interval}}=\frac{1}{2}\left[\begin{array}{cccc} 1 & \cos2\theta_{0} & \sin2\theta_{0} & 0\\ \cos2\theta_{0} & \cos^{2}2\theta_{0} & \cos2\theta_{0}\sin2\theta_{0} & 0\\ \sin2\theta_{0} & \cos2\theta_{0}\sin2\theta_{0} & \sin^{2}2\theta_{0} & 0\\ 0 & 0 & 0 & 0 \end{array}\right]\cdot \sum_{\theta =0^{\circ }}^{360^{\circ }}S\left(\theta \right)\left[\begin{array}{c} 1\\ \cos2\theta \\ \sin2\theta \\ 0 \end{array}\right]\\ & = & \sum_{\theta =0^{\circ }}^{360^{\circ }}\frac{S(\theta )}{2}\left[\begin{array}{c} 1+\cos2\theta_{0}\cos2\theta +\sin2\theta_{0}\sin2\theta \\ \cos2\theta_{0}+\cos^{2}2\theta_{0}\cos2\theta +\cos2\theta_{0}\sin2\theta_{0}\sin2\theta \\ \sin2\theta_{0}+\cos2\theta_{0}\sin2\theta_{0}\cos2\theta +\sin^{2}2\theta_{0}\sin2\theta \\ 0 \end{array}\right] \end{array} \end{array}$$

So the intensity transmitted by a polarizer can be derived from Equation 14, which is consistent with Equation 2.

(14) $$\begin{array}{ccc} I_{\theta_{0}} & = & \sum_{\theta =0^{\circ }}^{360^{\circ }}\frac{S(\theta )}{2}\cdot (1+\cos2\theta_{0}\cos2\theta +\sin2\theta_{0}\sin2\theta )\\ & = & \sum_{\theta =0^{\circ }}^{360^{\circ }}S\left(\theta \right)\cdot \cos^{2}(\theta_{0}-\theta )\\ & = & \sum_{\theta =0^{\circ }}^{360^{\circ }}S\left(\theta \right)\cdot p_{\theta_{0}}\left(\theta \right) \end{array}$$

Supposing:

- the directions are considered from 0° to 90° (i.e., 1/4 of a full interval) and the polarization states of multiple discrete directions are numerically approximated at 1° intervals (as the minimum integer angle, generally 1° is the angular resolution of the optical rotation device, and this numerical approximation is accurate enough for our purposes);
- the polarization states in each interval are approximately regarded as linear polarized with a specific electric field direction;

Then the intensities transmitted by discrete polarizer directions (*θ*_0_) and the intensities of polarized light with discrete electric field directions (*θ*) are related by:

(15) $${\left(I_{0},I_{1},\cdots ,I_{89},I_{90}\right)}^{T}=M\cdot {\left(S\left(0^{\circ }\right),S\left(1^{\circ }\right),\cdots ,S\left(89^{\circ }\right),S\left(90^{\circ }\right)\right)}^{T}$$

where a weight matrix M expressed by Equation 16 can be obtained according to Equation 2:

(16) $$M=\left[\begin{array}{ccccc} 1 & \cos^{2}1^{\circ } & \cdots & \cos^{2}89^{\circ } & 0\\ \cos^{2}1^{\circ } & 1 & \cdots & \cos^{2}88^{\circ } & \cos^{2}89^{\circ }\\ \vdots & \vdots & \ddots & \vdots & \vdots \\ \cos^{2}89^{\circ } & \cos^{2}88^{\circ } & \cdots & 1 & \cos^{2}1^{\circ }\\ 0 & \cos^{2}89^{\circ } & \cdots & \cos^{2}1^{\circ } & 1 \end{array}\right]=\left[\begin{array}{c} p_{0}\left(\theta \right)\\ p_{1}\left(\theta \right)\\ \vdots \\ p_{89}\left(\theta \right)\\ p_{90}\left(\theta \right) \end{array}\right]$$

On the one hand, for a certain row of M, $p_{\theta_{0}}\left(\theta \right)$ theoretically describes the contribution of each element *S*(*θ*) to the overall transmitted intensity $I_{\theta_{0}}$ by a polarizer. On the other hand, the difference between the rows of M can be regarded as the reason for the difference of captured polarization intensities. Thus, Equation 15 lays the foundation for the decomposition of the captured polarized intensities from the perspective of discrete electric directions, as well as for the refined PD characterization. However, Equation 15 actually has infinite solutions due to the rank of matrix M and augmented matrix (composed of M and column vector(*I*_0_,*I*_1_,⋅⋅⋅, *I*_89_,*I*_90_)^*T*^), so it can be difficult to solve the optimal solution directly, due to the high order of matrix M and large scale of image pixels. Therefore, this has been the motivation for us to study the indirect estimation method for discrete electric field intensity.

### Simulation Methods

In order to validate the advantage of the refined PD, simulations were implemented with a Polarized Monte Carlo simulation program. Generally, Polarized Monte Carlo sequentially traces photon's positions and states, and the total output Stokes vector is the sum of the product of each photon's normalized Stokes vector and weight.^[^ ^31^ ^]^ However, in order to obtain the polarized intensities of different electric field intervals, the photons with different electric field directions should be distinguished, in the range of 0° to 90°, at an interval of 5°(Δ*σ* = 5°), the polarization states of photons falling in each interval were counted respectively according to the *Q* component of the normalized Stokes vector, as shown in **Table** **1**. Then the photons’ backscattered Stokes vector in each interval was the sum of the product of each filtered photon's normalized Stokes vector (*I*, *Q*, *U*, *V*)^*T*^ and weight *W*.

**Table 1:** Discrimination of the photons with different electric field directions.

Specific direction *θ*	Normalized Stokes vector	Weight vector
0°	(1, 1, 0, 0)^*T*^	*p*_0_(*θ*)
5°	(1, cos 10°, sin 10°, 0)^*T*^	*p*_5_(*θ*)
…	…	…
85°	(1, cos 170°, sin 170°, 0)^*T*^	*p*_85_(*θ*)
90°	(1, −1, 0, 0)^*T*^	*p*_90_(*θ*)

Factors affecting the propagation of polarized photons were considered including particle size, medium density, wavelength and medium depth. As surgical smoke consists of particles with mean diameters ranging from 0.07 to 6.5 µm, influenced by the type of electrocauterized tissue and surgical device,^[^ ^1^, ^38^ ^]^ particle diameters of 0.2, 2.0, 6.0 µm were chosen for simulations and the densities (number of particles per cubic microns: *n*/µm^3^) were adjusted to appropriate values accordingly.^[^ ^39^ ^]^ The scattering coefficient and anisotropy *g* were calculated by calling a Mie function. The absorption coefficient was usually set to a low value^[^ ^40^, ^41^ ^]^ and the refractive indices of smoke particles and target (tissue) were selected according to refs. [42, 43]. The pencil beam illuminant was incident perpendicularly into the mixed medium and the receiving plane had a size of 25 × 25 cm, with 100 × 100 sampling points. The simulation parameters for the medium with large‐sized (6.0 µm) particles are shown in **Table** **2** and the simulated distributions for different densities are shown in Figure 2. The simulation parameters and distributions for the medium with small‐sized (0.2 µm) and medium‐sized (2.0 µm) particles are shown in the Supporting Information.

**Table 2:** Simulation parameters.

Parameters	Values
Particle diameter (µm)	6.0
Number of photons	50 000
Illumination polarization state	Horizontal linear polarized
Wavelength (nm)	630
Density (*n*/µm^3^)	0.8 × 10^−6^,1.0 × 10^−6^,1.2 × 10^−6^
Scattering coefficient (cm^−1^)	0.50, 0.62, 0.75
Absorption coefficient (cm^−1^)	0.01
Anisotropy *g*	0.73
Relative refractive index (smoke)	1.57+0.43i
Refractive index (surface)	1.50
Medium depth (cm)	8.0

### Prior‐Based Refined PD Estimation Method

The estimation of refined PD (Equation 3) requires estimating only the intensity of *S*(90°), however, due to the difficulty of its estimation in real‐world experiments, it is possible to approximate *S*(90°) based on prior information obtained from Polarized Monte Carlo simulations. According to Figure 2, *S*(90°) component was completely maintained in *I*_90_, however, if *S*(90°) was estimated only from *I*_90_, the result would probably be affected due to the existence of backscattered component (especially for strongly scattering media), so the impact of the backscattered component should be reduced from *I*_90_ before the estimation. Therefore, one prior‐based PD estimation solution was proposed, which included three steps:

- to reduce the backscattered component for a “clearer” orthogonal polarized channel based on multi‐channel cooperation;
- to approximate *S*(90°) component;
- to estimate the refined PD by Equation 3.

The first step could start by analyzing the distribution of $p_{\theta_{0}}\left(\theta \right)$ at certain directions *θ*_0_, based on the correlation between $p_{\theta_{0}}\left(\theta \right)$ and the captured polarization intensities. For active linear polarimetric imaging, according to the distribution of *p*_0_(*θ*), *p*_45_(*θ*) and *p*_90_(*θ*) in Figure 2g, *I*_90_ could reduce the impact of backscattered component, because the components with directions in the interval of 0° to 45° (especially those directions close to the incident polarization direction) contained most backscattered components, and only a small portion of the components were superimposed on *I*_90_, but still not that complete (partial backscattered component can still be found in Figure 2c). Therefore, from the perspective of artificially configured weight vectors $p_{\theta_{0}}\left(\theta \right)$, a target curve *p_T_* (*θ*) expressed by Equation 17 was expected to eliminate most of the backscattered components in orthogonal polarization, while almost maintain the complete components near *S*(90°), as shown by the black dashed line in Figure 2g.

(17) $$\left\{\begin{array}{c} p_{T}\left(\theta \right)=0,\theta \in \left[0^{\circ }\left.45^{\circ }\right)\right.\\ p_{T}\left(\theta \right)=\frac{\theta }{45}-1,\theta \in \left[45^{\circ }\right.\left.90^{\circ }\right] \end{array}\right.$$

Therefore, it was transformed into a curve fitting problem, by fitting the target curve *p_T_* (*θ*) (black dashed line) in Figure 2g, a weight vector for a “clearer” orthogonal polarization can be approximately generated. The base curves for the fitting were a series of $p_{\theta_{0}}\left(\theta \right)$ with variable amplitude, variable phase but fixed frequency, including *p*_0_(*θ*), *p*_45_(*θ*), *p*_90_(*θ*) in Figure 2g. The Matlab R2021a Curve Fitting Tool was adopted. According to our tests, base curves in at least any three directions were required for the fitting and the fitting performance did not improve when the number of base curves continued to increase. So the fitting equation could be expressed as:

(18) $$p_{F}\left(\theta \right)=a\cos^{2}\left(-\theta \right)+b\cos^{2}\left(45^{\circ }-\theta \right)+c\cos^{2}\left(90^{\circ }-\theta \right),\theta \in \left[0^{\circ }\right.\left.90^{\circ }\right]$$

where *a*, *b*, *c* were amplitude coefficients. The fitting result is shown by the orange dashed line in Figure 2g and the obtained amplitude coefficients were *a* = 0.5636, *b* = −0.8942, *c* = 1.395 (some fitting evaluation indices such as the sum of squares due to error (SSE = 0.0501), and coefficient of determination (R‐square = 0.9772) could also be obtained through fitting), which was related to the selection of the target curve and base curves. Then, we could use *p_F_* (*θ*) to replace *p*_90_(*θ*) and a “clearer” orthogonal polarization *I_crog_* that reduced the impact of backscattered component could be expressed as:

(19) $$\begin{array}{ccc} I_{crog} & = & \sum_{\theta =0^{\circ }}^{360^{\circ }}S\left(\theta \right)\cdot p_{F}\left(\theta \right)\\ & = & \sum_{\theta =0^{\circ }}^{360^{\circ }}S(\theta )\cdot \left[a\cos^{2}\left(-\theta \right)+b\cos^{2}\left(45^{\circ }-\theta \right)+c\cos^{2}\left(90^{\circ }-\theta \right)\right]\\ & = & a\cdot I_{0}+b\cdot I_{45}+c\cdot I_{90} \end{array}$$

where *I_crog_* was actually the sum of the product of the selected polarization intensities (corresponding to the base curves) and the amplitude coefficients.

The second step was to approximate *S*(90°) component. *I_crog_* reduced the impact of backscattered component while almost maintained the complete components near *S*(90°). In addition, according to the simulation results in Figure 2h, the distribution of *I_crog_* was flatter compared with *I*_90_ in Figure 2c, and the average intensity of *I_crog_* was at the similar level as *S*(90°). Therefore, *S*(90°) could be approximated as:

(20) $$S\left(90^{\circ }\right)\approx \frac{I_{crog}}{360}$$

The last step was to estimate the refined PD by Equation 3. Then the refined PD was used to replace the direct PD in Equation 1, forming the image restoration method based on refined PD.

### Experimental Methods

Ethical approval for the in vivo live mouse and in vivo human oral experiments was obtained from Zhejiang Lab Ethics Committee (reference number ZJSL‐2022‐9).

Experiments were designed and carried out with scenarios of ex vivo biological tissue, in vivo live mouse and in vivo human oral, in order to simulate the imaging through surgical smoke and capture a batch of real‐world biomedical polarized smoke data. Desktop and endoscopic integrated linear polarimetric imaging systems were built, respectively. For comparative experiments, the state‐of‐art model‐based and data‐based image restoration methods were adopted, as well as our proposed method, to process the polarized smoke images. The processed results were then evaluated and analyzed qualitatively and quantitatively. Details of the polarimetric imaging system are shown in the Supporting Information.

Ex vivo experiments for SpyderCheckr, biological samples of porcine kidney and liver on the desktop linear polarimetric imaging system were conducted, in order to preliminarily validate the effectiveness of the proposed method. After installing and calibrating the experimental equipment, the incident polarization state was set to horizontal linear polarized (0°), before the smoke injection, clear images of the sample (without scattering media) were taken in advance as references. As the smoke was released manually through the fogger until the sample was completely obscured, then four linear polarized channels were continuously collected at a frame rate of around 5 frames per second. The full‐reference image quality assessment indicators PSNR, SSIM, CIEDE2000 were adopted for the quantitative analysis.

The polarimetric imaging experiments using mouse tissue as the target and paired with a polarization maintaining endoscopic imaging system, a laparoscopic simulator could more closely simulate the surgical scenario, thus providing a reference for the practical application. The experiment was carried out on 18th April 2023 at Zhejiang Lab. After calibrating the parameters such as initial polarization state, intensity, white balance, and focal length, the linear polarimetric endoscopic imaging system was fixed and positioned in the umbilical access of the simulator, while the tools such as high‐frequency soldering iron, needle holder, and scissors were placed on both sides of the simulator access. A white mouse was anaesthetised, fixed, sterilized, then its chest cavity was opened and then placed on a carrier table inside the simulator.

The first round of experiment was to ensure the integrity of the mouse sample before and after the release of the smoke (target consistency), so that ground truth could be captured and used as a quality assessment criterion for the restored images. Horizontal linear polarized (0°) was set as the incident polarization state, and smoke was generated by cutting a piece of fat tissue around the mouse with a high‐frequency soldering iron and the polarized images were continuously acquired in the smoke environment. The validation experiments with the USAF‐1951 Resolution Target for spatial resolution restoration followed similar experimental procedures as well. The acquired polarization information was directly processed and a full‐reference quantitative evaluation (PSNR, SSIM, CIEDE2000) of the processed results was performed, without performing operations such as color correction to ensure accurate display of polarization information.

In order to simulate the operation more realistically, in the second round of experiments, surgical instruments were used to simulate “operating” on mouse and smoke was created, capturing 1 frame per 1 s and ensuring that the mouse tissues and surgical instruments were covered by smoke simultaneously so as to validate the recovery performance of our method. Because the manipulation of the surgical instruments caused the scenario to change constantly and prevented us from obtaining the corresponding ground truth. Therefore, the non‐reference image quality assessment indicators BIQI, ILNIQE, and BRISQUE were adopted for the quantitative analysis.

Polarization images of oral tissues containing smoke were captured to meet the in vivo imaging requirements where tissues and smoke coexist. The experiment was carried out on 17th April 2023 at Zhejiang Lab. First, the linear polarimetric endoscopic imaging system was adjusted and calibrated, then after the subject inhaled smoke, the imaging system was immediately placed in the mouth and polarized images were continuously captured. Similarly, ensuring stillness of oral or tongue tissues during the experiment posed a challenge, preventing the acquisition of corresponding ground truth for quantitative evaluation. Therefore, BIQI, ILNIQE, and BRISQUE were utilized for non‐reference quantitative analysis.

### Comparative Restoration Methods

Several model‐based and data‐based image restoration methods were chosen for comparison. Two classic model‐based methods, dark channel prior based and haze line prior based methods, have been proven to be effective in previous work.^[^ ^26^ ^]^ In recent years, scholars have proposed numerous data‐based methods, FFA‐Net,^[^ ^16^ ^]^ DehazeFormer,^[^ ^17^ ^]^ C2PNet,^[^ ^18^ ^]^ and DEA‐Net^[^ ^19^ ^]^ were chosen as the comparative methods due to their outstanding performance. Because i) polarization data are all real‐world data obtained in experiments (cannot be synthesized), ii) polarization data can be affected by many influencing factors, making it difficult to collect a comprehensive polarization dataset for training the above network architecture. Therefore, the pre‐trained models of data‐based methods were directly adopted to validate their generalization abilities. It is worth emphasizing that the above methods usually provide pre‐trained models specifically for different datasets (such as indoor, outdoor, etc.), so after testing, the pre‐trained models with better performance for different experimental scenarios were selected, as shown in **Table** **3**.

**Table 3:** The adopted data‐based methods and corresponding pre‐trained models.

Data‐based method**s**	Adopted pre‐trained models
FFA‐Net (AAAI 2020)	OTS (for ex vivo and in vivo)
DehazeFormer (IEEE TIP 2023)	Outdoor ‐m (for ex vivo and in vivo)
C2PNet (CVPR 2023)	OTS (for ex vivo and in vivo)
DEA‐Net (IEEE TIP 2024)	OTS (for ex vivo) and HAZE4K (for in vivo)

## Conflict of Interest

The authors declare no conflict of interest.

## Author Contributions

J.Q. and D.S.E. conceived the project. D.W., D.S.E., J.G., and J.Q. conceptualized and refined the methodology. D.W. and J.Q. designed and performed tissue experiments, J.S. and D.W. built and commissioned experimental devices for the in vivo experiments, J.Q., D.W., and J.S. performed the in vivo experiments. D.W. performed simulation and experimental data processing and analysis. D.W., J.Q., and D.S.E. wrote the manuscript. All the authors reviewed and commented on the manuscript.

## Supporting information

Supporting Information

## Data Availability

The data that support the findings of this study are available from the corresponding author upon reasonable request.
