# Supplementary material for: Computational Polarization Imaging In Vivo through Surgical Smoke Using Refined Polarization Difference
Source: Adv Sci (Weinh). 2024 Jun 5;11(30):2309998. doi: 10.1002/advs.202309998 (PMC11321673; doi:10.1002/advs.202309998)
Supplement: Supplementary file 1 — Supporting Information [file ADVS-11-2309998-s001.docx]

Supporting Information

**Computational polarization imaging *in vivo* through surgical smoke using refined polarization difference**

### *Daqian Wang, Jiawei Song, Jun Gao, Ji Qi^*^ and Daniel S. Elson*

**1. Polarized Monte Carlo modeling containing medium and target**

Monte Carlo method is recognized as a flexible yet rigorous approach for simulating photon transport in scattering media,^[28]^ and has been widely applied to tasks requiring high precision such as inversion of optical properties of scattering media,^[28]^ calibration of scattering phantom,^[29]^ radiation dose calculation in radiotherapy.^[30]^ In our study, Polarized Monte Carlo modeling was employed to study the propagation of polarized photons in the environment, laying the groundwork for the development and validation of our proposed method. To ensure that the simulation results align with actual imaging results, efforts have been made in the following aspects:

i) Modeling method: we aimed to address the complexity of imaging conditions, which involve both the imaging target and the smoke medium. Currently, existing models employ often simplified methods when dealing with polarized light and interactions between the smoke particles and target surfaces (such as specular reflection, diffuse reflection). This can lead to inaccuracies in modeling complex environments. Therefore, we enhanced classical polarized Monte Carlo algorithms^[31-37]^ by integrating modules for polarimetric scattering, rough surface geometry, and polarimetric reflection, without altering the algorithms governing the interaction between polarized photons and the particles or surfaces. This integration allows our model to be applied to a wider range of scenarios. We performed a single-step debugging process to test the accuracy and reliability of the code.

ii) Simulation parameter settings: all our parameter configurations are supported by relevant literature, as detailed in Section 5, Simulation Methods.

iii) The simulation results aligns with the theoretical analysis, as detailed in the discussion of the Polarized Monte Carlo simulation results.

Our proposed method has proven effective across various experimental scenarios, validating the reliability of prior knowledge and Polarized Monte Carlo simulation.

Specifically, the modeling of polarimetric scattering module can refer the method from ref. [31] with source code from ref. [32], including an update of the photon movement state and polarization state after scattering. The modeling of rough surfaces geometric module can refer the method from refs. [33-34] with source code from ref. [35], including the determination of a photon’s reflection direction. The modeling of polarimetric reflection module can refer the method from ref. [36] with source code from ref. [37], including the determination of a reflected photon’s Mueller Matrix and updated of polarization state.

The algorithm execution process was as follows: after setting up the parameters of smoke medium, target surface and light conditions respectively, the photons were launched and then their propagation process (scattering, reflection) were been tracked. More importantly, the variation of photon polarization state after each movement was updated. Finally, the state and energy of those photons received by the detector were counted.

We conducted simulations for the medium with small-sized (0.2 μm) and medium-sized (2.0 μm) particles (falling within the range typical for surgical smoke), as a complement to the simulation experiment depicted in Figure 2. The parameter settings are shown in Table S1, and the results are presented in Figure S1. A consistent downward trend of $S(\theta)$ was observed with increasing $\theta$. For the medium with small-sized particles, where the scattering coefficient was low, polarized photons underwent few scattering events, resulting in the backscattered light’s polarization state closely resembling that of the incident light, leading to a concentrated distribution of $S(\theta)$. Conversely, the concentration of energy distribution of $S(\theta)$ decreased with increasing scattering coefficient, as depicted in Figure S1a,d.

In addition, $p_{0}(\theta)$ varied from 1 to 0 in the interval of 0° to 90°, as shown in Figure 2g, so $I_{0}$contained a large amount of backscattered components closely aligned with the incident polarization state, representing the most affected polarized channel by these components, as shown in Figure S1b,e. $I_{90}$ exhibited a relatively uniform distribution with $\theta$ variation, as shown in Figure S1c,f. When intervals approached 0° or 90°, $I_{90}$ decreased due to low values of $p_{90}(\theta)$ or $S(\theta)$, potentially leading to a peak in the middle interval with the highest $I_{90}$. Thus, the distribution of $I_{90}$ initially rose and then declined, as shown in Figure S1f. These simulated outcomes aligned with theoretical analyzes. In scenarios where comprehensive quantitative investigations of all factors are impractical via experimental means, Polarized Monte Carlo presents a feasible approach for studying polarized transmission characteristics.

Table S1. Simulation parameters

| Parameters | Values | |
| --- | --- | --- |
| Particle diameter (μm) | 0.2 | 2.0 |
| Number of photons | 50000 | 50000 |
| Illumination polarization state | Horizontal linear polarized | Horizontal linear polarized |
| Wavelength (nm) | 630 | 630 |
| Density$\text{(}n/\text{μm}^{3})$ | $2.0\times10^{\text{-3}}$,$3.0\times10^{\text{-3}}$,$4.0\times10^{\text{-3}}$ | $6.0\times10^{\text{-6}}$,$7.0\times10^{\text{-6}}$,$8.0\times10^{\text{-6}}$ |
| Scattering coefficient$\text{(cm}^{\text{-}1})$ | 0.17, 0.26, 0.35 | 0.55, 0.64, 0.74 |
| Absorption coefficient$\text{(cm}^{\text{-}1})$ | 0.01 | 0.01 |
| Anisotropy *g* | 0.21 | 0.74 |
| Relative refractive index (Smoke) | 1.57+0.43i | 1.57+0.43i |
| Refractive index (Surface) | 1.50 | 1.50 |
| Medium depth (cm) | 8.0 | 8.0 |


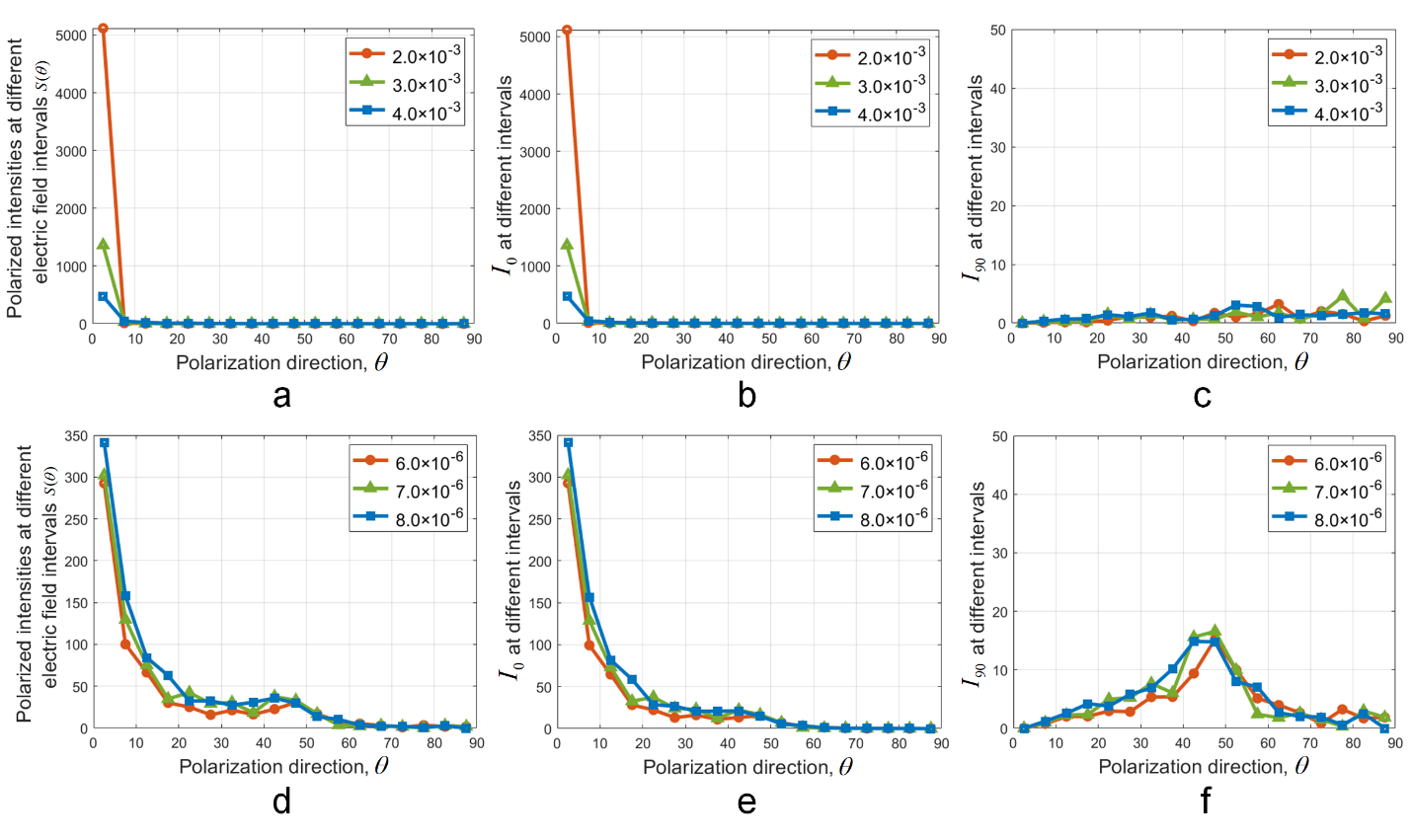


**Figure S1.** Variation of simulated distributions for the medium with small-sized (0.2 μm) particles. a) $S(\theta)$, b) $I_{0}$, c) $I_{90}$; and medium-sized (2.0 μm) particles: d) $S(\theta)$, e) $I_{0}$, f) $I_{90}$.

**2. Linear polarimetric imaging system**

We built a benchtop and an endoscopic linear polarimetric imaging systems for *ex vivo* and *in vivo* experiments, respectively. The benchtop system was used to capture the real-world smoke-affected polarized images of *ex vivo* targets, as shown in Figure S2a. The polarization state analyzer (PSA) was a LUCID-TRI050S polarization camera with Sony polarized sensor IMX250MYR allowing four linear polarized channels to be captured simultaneously in color. The polarization state generator (PSG) consisted of a white light illuminant GI-0604 and a calibrated linear polarizer. All the equipment was placed in a closed container, and a fogger AB-900 injected a controllable level of fog (from heated fluid) through a pipe to simulate surgical smoke.


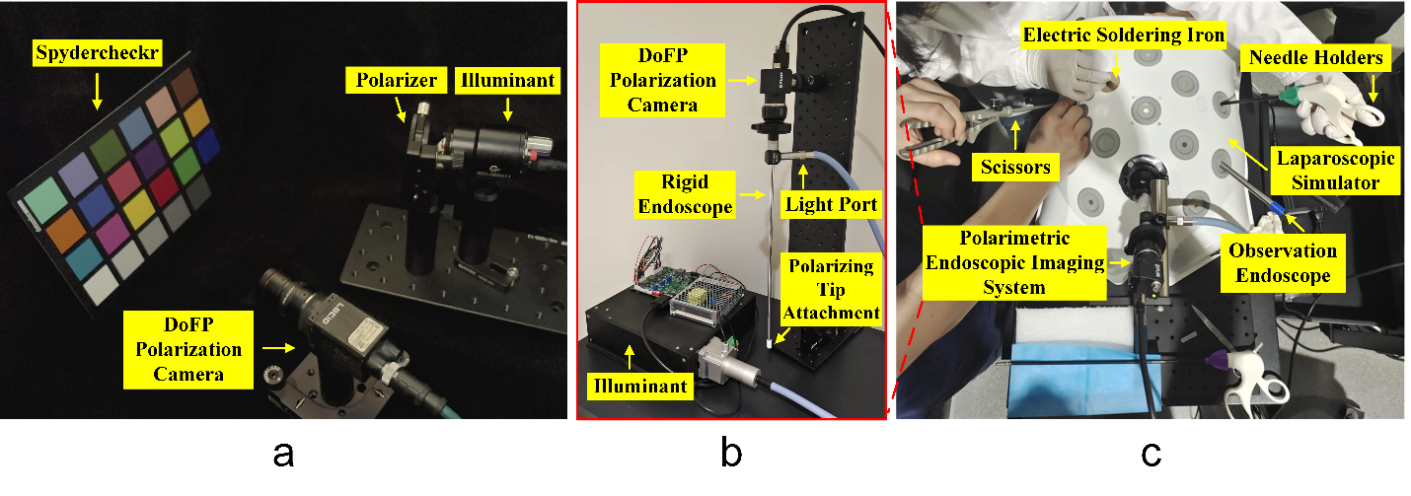


**Figure** **S2.** The linear polarimetric imaging system. a) Desktop system, b) Polarimetric endoscopic imaging system, c) Polarimetric endoscopic imaging system mounted in a laparoscopic simulator

A unique linear polarimetric endoscopic imaging system^[22]^ was built for *in vivo* experiments, as shown in Figure S2b,c. The system contained a polarization maintaining rigid endoscope (COMEG, 6 mm diameter, 0° view angle) with a 3D-printed rotatable polarizing tip attachment (8 mm outer diameter, 1 cm length, held a ring-shaped wideband linear polarizing plate (XP44-40) over the illumination channel at the distal end of the endoscope to polarize the illumination light), a FLIR Blackfly BFS-U3-51S5PC polarization camera with Sony polarized sensor IMX250MYR (exposure time was 0.1s, frame rate was 8 frames per second, gain and gamma were 0), as well as an adjustable illuminant Rayfine RFFM-16A5 (white light illumination). In order to accurately simulate the surgical imaging conditions, we also equipped a set of biomimetic laparoscopic simulator (LapGame), where the polarization imaging endoscope was placed at the umbilical access, the high-frequency electric soldering iron (DELIXI 203H 90W, used to simulate the surgical energy-generating instrument), the needle holder and scissors (to simulate the surgical tools) were placed at the side entrance of the simulator. The imaging sample was placed inside the simulator, where the smoke could be generated by cutting the biological tissue, and the smoke-affected polarized data was continuously collected at a rate of 1 frame per second.

**3. *In vivo* mouse experiment involving motion**

Intraoperative conditions involve constant motion, including tissue movement, instrument manipulation, smoke flow, etc., which are common and unavoidable. In order to demonstrate the performance of our method under these various motion artifacts, we present three typical images for restoration. For tissue movement caused by instrument manipulation under relatively static smoke, we designated this as Group 1; for tissue movement resulting from instrument manipulation under flowing smoke with different densities, as Group 2, while Figure 6 (moment 2) in manuscript illustrates the processing result of relatively stationary tissue under flowing smoke.

Qualitative and quantitative results indicated that the performance of various methods was similar to that of the previous sets. Both blurry (in motion) and clear (stationary) targets, obscured by smoke (whether flowing or static), were successfully restored using the proposed method, as illustrated in Figure S3. This success stemmed from the enduring applicability of the polarization-based image degradation model in the presence of motion artifacts. The captured polarimetric intensities could be divided into the attenuated intensity from the target and scattered ambient light, as described in Section 5. Time was not a parameter within the model, thus rapid movement of targets or smoke flow did not affect the validity of the model. Despite losing local details and structural information, the information within these areas was also encoded in the corresponding four linear polarization intensities. The effective transmission rate and target intensity, accounting for the motion of both the smoke and the target, could be sequentially estimated on a pixel-wise basis from the experimentally obtained polarization difference. However, the restored images with motion artifacts may pose challenges for further medical analysis and diagnosis.


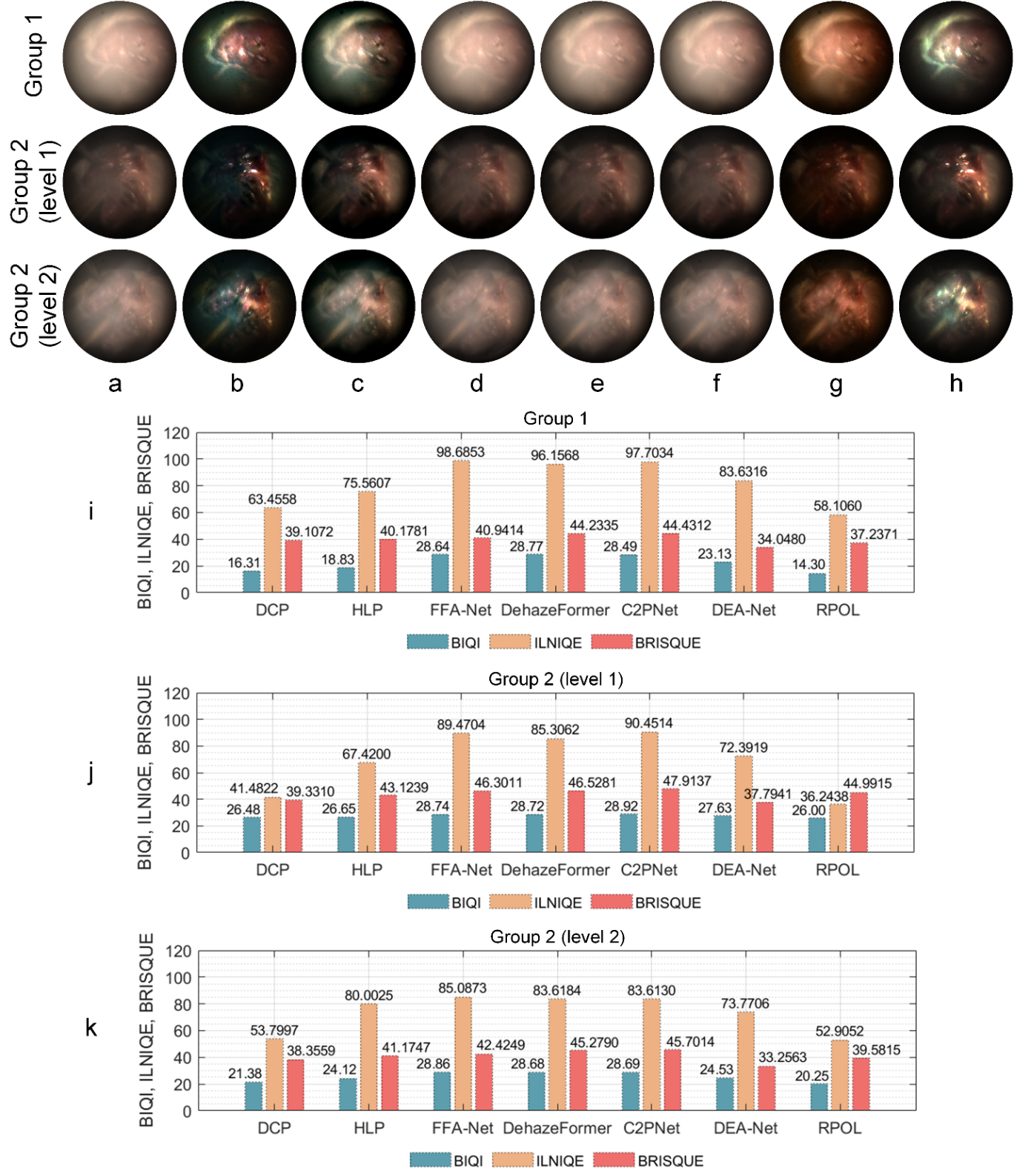


**Figure S3.** Comparison of smoke removal results for motion artifacts across different methods (Group 1: for tissue movement caused by instrument manipulation under relatively static smoke; Group 2: for tissue movement resulting from instrument manipulation under flowing smoke with different density levels). a) 0° polarized smoke images, and results processed by b) DCP, c) HLP, d) FFA-Net, e) DehazeFormer, f) C2PNet, g) DEA-Net, and h) RPOL, with further quantitative results in i-k) BIQI, ILNIQE, and BRISQUE (the lower the better).

**4. Assessing the performane for spatial resolution restoration**

Due to the need for surgeons to observe and identify delicate structures such as blood vessels, nerves, and tissue boundaries during surgery to ensure precise positioning and manipulation, the imaging experiment utilized the USAF-1951 Resolution Target to validate the method’s spatial resolution restoration capability. A ceramic target, with precision of $\pm3\text{ μm}$ and thickness of 1 mm, was chosen for the experiment. After configuring the imaging system, the target was fixed in the laparoscopic simulator, and smoke-free ground truth images were captured for reference. Subsequently, smoke was generated by cutting a piece of fat tissue with a high-frequency electric soldering iron, and smoke images were continuously acquired using the linear polarimetric endoscopic imaging system.

The ground truth, smoke images at different densities, and the restoration results of various methods for the Resolution Target’s Group 0 to Group 3 patterns are shown in Figure S4. The point-like objects visible on the target included tiny specular reflection points on the ceramic surface, as well as oil droplets and smoke particles adhered during smoke generation. Qualitative analysis of the target in columns 1 and 3 (Group 0 and Group 1) and columns 2 and 4 (Group 2 and Group 3) in Figure S4 revealed that the resolution of the ground truth and smoke images was approximately 2.0 lp/mm (Group 3 Element 1) and in the range of 1.59-1.78 lp/mm (Group 2 Element 5-6), respectively.

Various image restoration methods were applied to process smoke images with two different densities. Due to space limitations, FFA-Net, DehazeFormer, and C2PNet were not included, as their resolution restoration effects relative to the smoke images were not significant. The four listed methods all demonstrated resolution restoration effects, as quantified in Table S2. Notably, our proposed RPOL method exhibited superior image restoration and contrast enhancement effects, leading to an improvement in spatial resolution (in the range of 1.78-2.0 lp/mm). Furthermore, we provided quantitative results of SSIM and PSNR for each group of processed images for reference. These metrics assess the similarity between the restored and reference images, however, they may not entirely capture the quality of spatial resolution restoration.


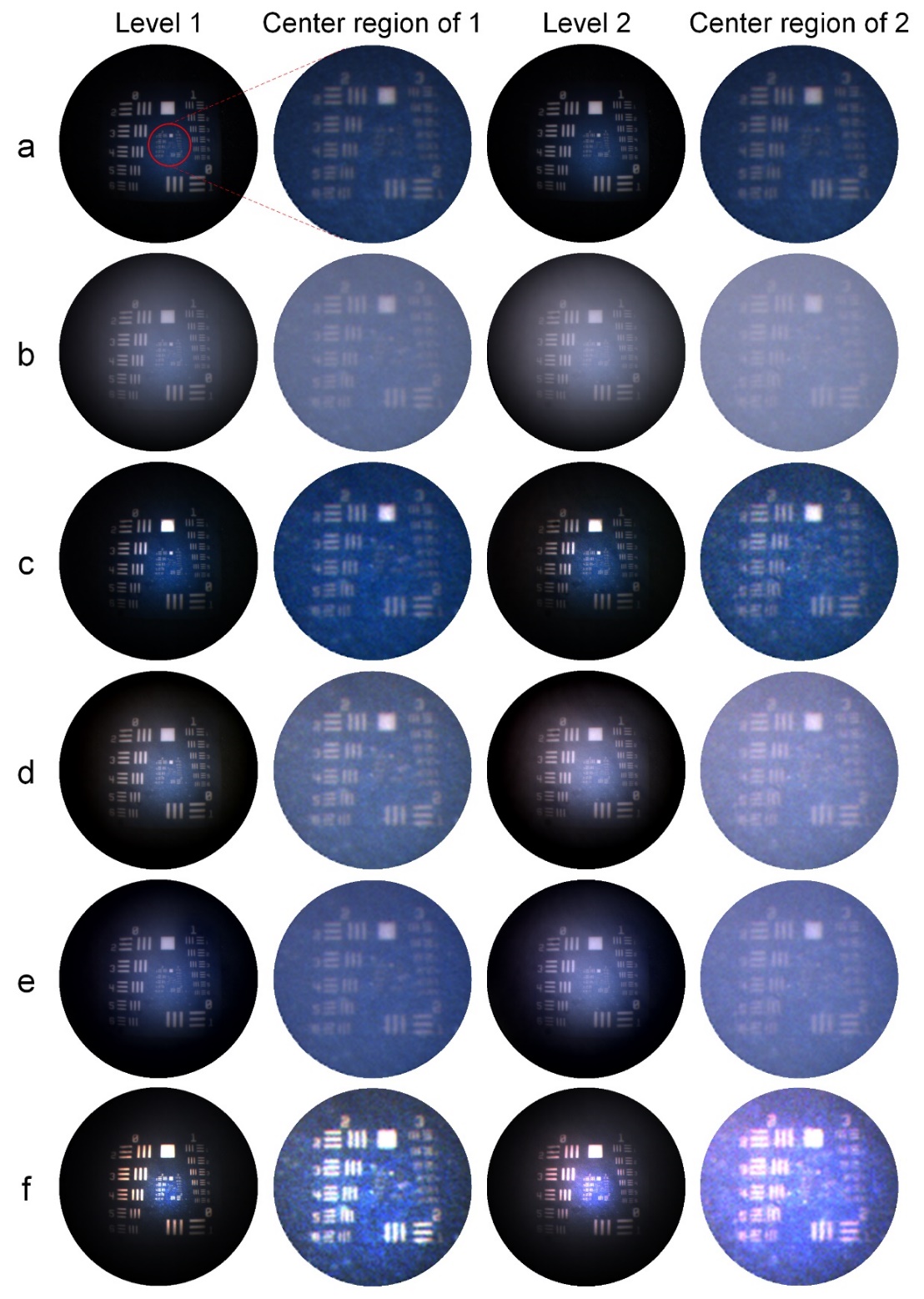


**Figure S4.** Comparison of smoke removal results with USAF-1951 Resolution Target for different smoke densities (Level 2 was denser) across different methods. For better visualization, the center region of the Resolution Target (Group 2 and 3) was enlarged and displayed next to the corresponding image. a) Co-polarized ground truth b) Co-polarized smoke images, and results processed by c) DCP, d) HLP, e) DEA-Net, and f) RPOL. The results processed by FFA-Net, DehazeFormer, and C2PNet were not listed as the resolution restoration was less pronounced compared to smoke images.

Table S2. Quantitative evaluation of spatial resolution restoration (the higher the better).

| Level | Index | DCP | HLP | DEA-Net | RPOL |
| --- | --- | --- | --- | --- | --- |
| 1 | Resolution (lp/mm)  SSIM  PSNR | 1.78-2.00  0.9100  30.53 | 1.78  0.8047  22.50 | 1.78  0.8952  26.14 | 1.78-2.00  0.8232  22.26 |
| 2 | Resolution (lp/mm)  SSIM  PSNR | 1.78-2.00  0.8229  27.24 | 1.78  0.7337  18.29 | 1.78  0.7763  20.16 | 1.78-2.00  0.7065  18.91 |
